# SCYL1 deficiency in CALFAN syndrome is associated with ER stress and cell death

**DOI:** 10.1242/dmm.052371

**Published:** 2025-11-27

**Authors:** John Hellicar, Tal Dattner, Tian Sun, Lily Percival, Ruby Chrisp, Andrea Pietrobattista, Tomasz Witkos, Aleksander Mironov, Lina Leghlam, Carolin Jentsch, Stefan Koelker, Georg F. Hoffmann, Christian Staufner, Wanjin Hong, Dominic Lenz, Martin Lowe

**Affiliations:** ^1^ Division of Molecular and Cellular Function, School of Biological Sciences, Faculty of Biology, Medicine and Health, University of Manchester, The Michael Smith Building, Oxford Road, Manchester M13 9PT, UK; ^2^ Heidelberg University, Medical Faculty Heidelberg, Center for Pediatric and Adolescent Medicine, Department I, Division of Pediatric Neurology and Metabolic Medicine, 69120 Heidelberg, Germany; ^3^ Division of Metabolic Diseases and Hepatology, Bambino Gesù Children's Hospital IRCCS, 00165 Rome, Italy; ^4^ Electron Microscopy Core Facility, Faculty of Biology, Medicine and Health, University of Manchester, The Michael Smith Building, Oxford Road, Manchester M13 9PT, UK; ^5^ Institute of Molecular and Cell Biology, Agency for Science, Technology and Research (A(∗)STAR), 61 Biopolis Drive, Proteos, Singapore 138673, Singapore

**Keywords:** SCYL1, CALFAN syndrome, ER stress, Procollagen, Secretory pathway, Golgi apparatus

## Abstract

CALFAN syndrome is a rare genetic disorder affecting the nervous system and liver, with skeletal abnormalities also reported. It is caused by mutations in *SCYL1*, a gene encoding a ubiquitously expressed protein localized to the secretory pathway. SCYL1 interacts with trafficking components, including ARF GTPases and the COPI vesicle coat complex, and appears to function in retrograde secretory trafficking. Despite this knowledge, the mechanisms that underlie CALFAN pathology remain poorly understood. Here, using fibroblasts obtained from patients diagnosed with CALFAN syndrome and from SCYL1 knockout fibroblasts, we reveal an accumulation of the abundant secretory cargo procollagen type I in the endoplasmic reticulum (ER) upon SCYL1 deficiency. Surprisingly, we failed to observe procollagen-I-trafficking defects in the SCYL1-deficient cells. Nevertheless, ER accumulation of procollagen-I correlated with ER distension and induction of ER stress in the patient fibroblasts, which also underwent increased cell death. The phenotypes were observed at elevated temperatures, mimicking the induction of pathology under febrile conditions in patients with CALFAN syndrome. Our data suggest that ER stress induction is a pathological mechanism in CALFAN syndrome and that targeting this process may represent a therapeutic strategy.

## Teaser

**Summary:** Fibroblasts obtained from patients with CALFAN syndrome and who are deficient in the Golgi protein SCYL1, accumulate procollagen in the ER, and undergo ER stress and cell death. Our results suggest that ER stress is a pathological mechanism in CALFAN syndrome.

## INTRODUCTION

Deficiency of the SCYL1 protein causes a rare autosomal recessive syndromal disorder characterized by low to mildly elevated gamma-glutamyltransferase (GGT) cholestasis, recurrent episodes of acute liver failure and neurodegeneration (CALFAN) syndrome [see Online Mendelian Inheritance in Man (OMIM) #616719] (Lenz et al., 2018; McNiven et al., 2021). Biallelic pathogenic variants in *SCYL1* were first described in three human individuals in 2015 causing recurrent acute liver failure, spinocerebellar ataxia and peripheral neuropathy (Schmidt et al., 2015). To date, 23 affected individuals have been described in the literature with a predominant hepatological and neurological phenotype (Chavany et al., 2020; Incecik et al., 2018; Isa et al., 2023; Lenz et al., 2018; Li et al., 2019; McNiven et al., 2021; Peters et al., 2025; Schmidt et al., 2015; Shohet et al., 2019; Smith et al., 2017; Spagnoli et al., 2019; Yigit et al., 2022; Youssef et al., 2023). While the hepatic phenotype presents with acute liver failure with cholestasis and a low GGT activity with progressive liver remodeling over time, the neurological phenotype is variable, ranging from secondary microcephaly only to a pronounced neurological phenotype involving the central and peripheral nervous system (Incecik et al., 2018; McNiven et al., 2021; Schmidt et al., 2015; Youssef et al., 2023; Chavany et al., 2020; Isa et al., 2023; Lenz et al., 2018; Li et al., 2019; Peters et al., 2025; Shohet et al., 2019; Smith et al., 2017; Spagnoli et al., 2019; Yigit et al., 2022). Interestingly, in ten patients a skeletal phenotype has been observed (Lenz et al., 2018; Li et al., 2019; Peters et al., 2025; Shohet et al., 2019; Smith et al., 2017; Spagnoli et al., 2019). Pathogenic variants have been identified throughout the *SCYL1* gene and, to date, no genotype−phenotype correlation has been revealed.

SCYL1 is a member of the SCY-like family of proteins that contain an N-terminal kinase-like domain followed by a variable number of Huntingtin, elongation factor 3, the A subunit of protein phosphatase 2A (PP2A) and the signaling kinase TOR1 (HEAT) repeats that mediate oligomerization, and variable C-terminal regions that mediate protein interactions (Pelletier, 2016). SCYL1 is localized to the ER−Golgi intermediate compartment (ERGIC) and Golgi apparatus, where it can interact with both ARF GTPases and the COPI vesicle coat complex, which binds via a C-terminal RKXX motif (Burman et al., 2008, 2010; Hamlin et al., 2014; Witkos et al., 2019). A pool of SCYL1 is also present at the *trans*-Golgi network (TGN), which interacts with the coiled-coil protein GORAB (Witkos et al., 2019). SCYL1 is thought to form a scaffold for ARF and COPI that can stabilize COPI assembly, and promote formation of COPI-transport vesicles at the ERGIC and Golgi membrane (Hamlin et al., 2014; Witkos et al., 2019). In *Drosophila*, the SCYL1 orthologue YATA has been shown to play a major role in COPI localization (Saito et al., 2021). However, despite being one of the major COPI interaction partners in cells, depletion or knockout (KO) of SCYL1 has only a modest effect on protein trafficking in the secretory pathway, with reduced Golgi to ER retrograde trafficking, which is accompanied by subtle changes in Golgi morphology (Burman et al., 2008, 2010; Kaeser-Pebernard et al., 2022). It has also recently been shown that SCYL1 is an mTORC1 substrate, where phosphorylation is required to maintain its Golgi localization (Kaeser-Pebernard et al., 2022). SCYL1 dephosphorylation redistributes the protein to endolysosomal compartments, where it may participate in exosome secretion. SCYL1 has also been implicated in nucleocytoplasmic shuttling of tRNA (Chafe and Mangroo, 2010) and a splice variant has been reported to localize to the centrosome (Kato et al., 2002), the functional significance of which is unclear.

It is currently unclear how loss of SCYL1 results in the CALFAN patient phenotype. SCYL1-deficient mouse models have a prominent neurological phenotype but fail to recapitulate the liver phenotype of CALFAN syndrome (Kuliyev et al., 2018; Pelletier et al., 2012). Mouse models have also shown that SCYL1 and SCYL3 – a homologue that can also bind COPI – share overlapping function in maintaining motor neuron health, but the mechanisms remain unclear (Kuliyev et al., 2018). Here, we have explored the effects SCYL1 deficiency has on the secretory pathway by using dermal fibroblasts derived from patients with CALFAN syndrome. Our findings indicate an accumulation of secretory cargo in the endoplasmic reticulum (ER) that is accompanied by disruption of ER homeostasis and ER stress. These phenotypes are exacerbated at high temperatures, i.e. 40°C and 42°C, mimicking the febrile state of patients with CALFAN. Our results provide new insight into the pathomechanism of CALFAN syndrome and suggest that ER stress is a major contributor to the patient phenotype.

## RESULTS

### Patient genotype, phenotype and respective protein levels of SCYL1

To date, 23 individuals with CALFAN syndrome have been described, carrying 17 different variants distributed throughout the *SCYL1* gene (Incecik et al., 2018; McNiven et al., 2021; Schmidt et al., 2015; Youssef et al., 2023; Chavany et al., 2020; Isa et al., 2023; Lenz et al., 2018; Li et al., 2019; Shohet et al., 2019; Smith et al., 2017; Spagnoli et al., 2019; Yigit et al., 2022) (Fig. S1). The identified variants comprise missense and nonsense mutations, as well as splice site variants and deletions. Notably, a limited number of patients share the same genotypic configuration, and no discernible clustering of variants affecting specific protein domains was observed. Variants in bold specifically illustrate the variants derived from fibroblasts utilized in this study (Fig. S1). All four patients and their corresponding genotypes have been reported before (Lenz et al., 2018). Reduced SCYL1 protein levels (compared to that of wild type) have previously been reported in three of the four patient fibroblasts (31.7% in CALFAN_1, 16.0% in CALFAN_2 and 7.1% in CALFAN_5) (Lenz et al., 2018). For CALFAN_4 SCYL1 protein abundance was also found to be reduced (data not shown). Clinical manifestations in these four individuals were characterized by hepatic and neurological phenotypes across all four patients (Table S1) (Lenz et al., 2018). Skeletal abnormalities and musculature involvement were noted in two of these cases. Intriguingly, growth impairment and endocrine system anomalies were absent in all four patients.

### SCYL1 deficiency causes increased ER levels of procollagen

**Fig. 1.**
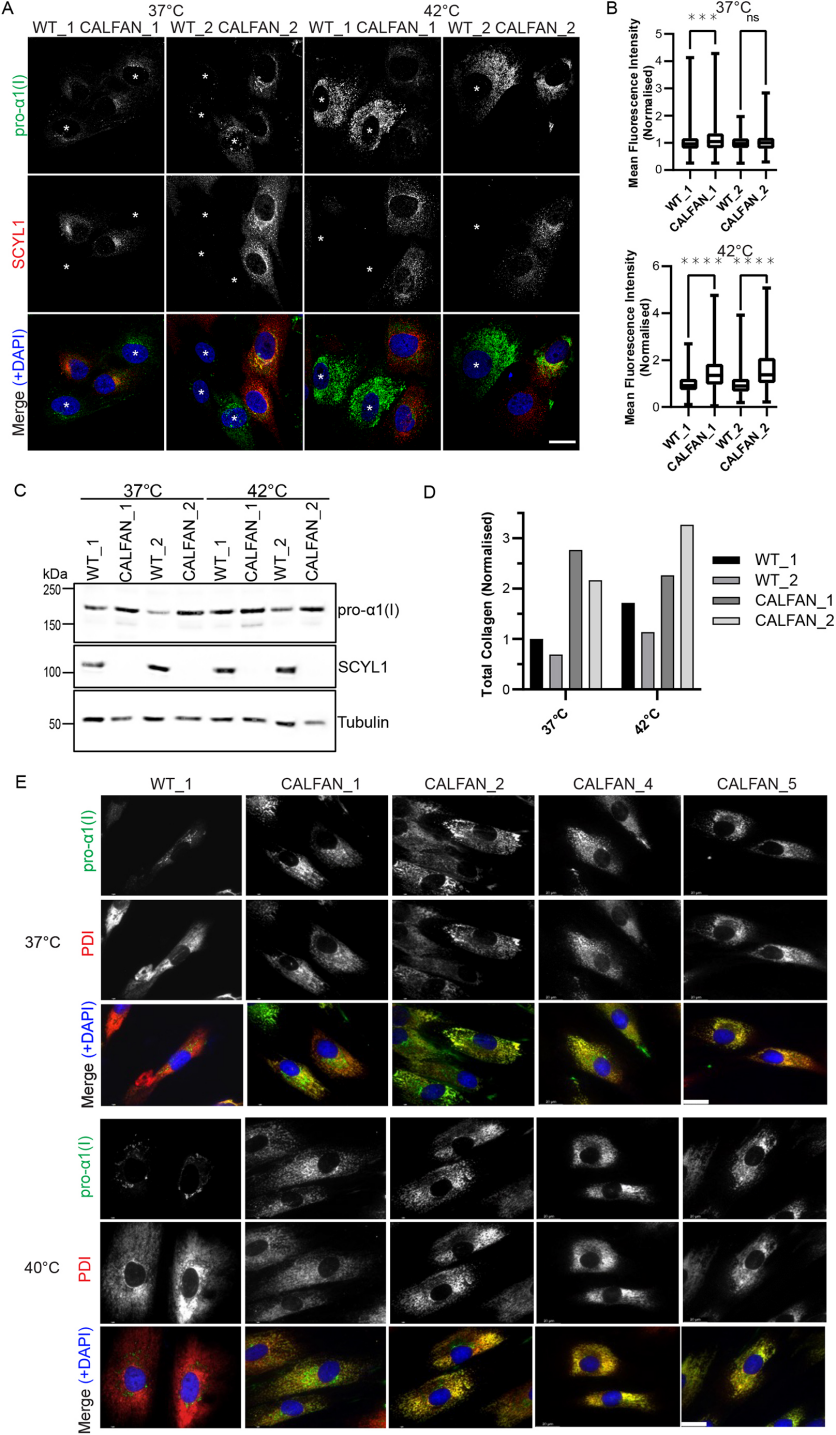
**Analysis of procollagen I abundance in CALFAN fibroblasts.** (A) Immunofluorescence microscopy images showing different wild-type (WT; i.e. WT_1 or WT_2) and CALFAN fibroblasts (i.e. CALFAN_1 or CALFAN_2) grown together as indicated for two days in medium containing 0.25 mM ascorbic acid. For the final 24 h cells were either kept at 37°C or shifted to 42°C prior to fixation and labelling with antibodies against procollagen I pro-α1 (I) (green) and SCYL1 (red). Asterisks denote CALFAN fibroblasts. (B) Mean fluorescence intensity of pro-α1(I) in cells grown as described for in A. Box and whiskers plot showing max and min values, *n*=3 independent experiments, with ≥50 cells of each cell type analyzed per experiment. Mann–Whitney test; ns, not significant; ***P<0.001, ****P<0.0001. (C) Western blot of wild-type (WT) and CALFAN cells grown for a total of 2 days in the presence of 0.25 mM ascorbic acid. Cells were cultured at 37°C or 42°C for the final 24 h as indicated. Samples were blotted by using antibodies against pro-α1(I), SCYL1 and tubulin; the latter was used as loading control. (D) Quantification of the western blot results shown in C, with procollagen I levels normalized to tubulin. (E) Immunofluorescence microscopy images of wild-type or CALFAN fibroblasts grown at 37°C or 40°C without ascorbic acid for 24 h prior to fixation and labelling with antibodies against pro-α1 (I) (green) and PDI (red). Scale bars, 10 µm.

Because SCYL1 is localized to the early secretory pathway and interacts with ARF GTPases and COPI, both of which are important for secretory trafficking (Burman et al., 2008, 2010; Hamlin et al., 2014; Witkos et al., 2019), we assessed whether loss of SCYL1 in CALFAN cells affects abundance and subcellular distribution of secretory cargo proteins. We used dermal fibroblasts isolated from patients with CALFAN syndrome and analyzed procollagen type I as a major secreted protein in this cell type. Initial experiments used fibroblasts from two healthy individuals (wild-type control) and two patients with CALFAN (CALFAN_1 and CALFAN_2), which were cultured at 37°C or at an elevated temperature of 42°C to mimic the febrile state that induces liver pathology in patients. The fibroblasts were cultured in the presence of ascorbic acid, which promotes the synthesis of procollagen I (Murad et al., 1981), and wild-type and CALFAN fibroblasts were co-cultured to allow a direct comparison between the two cell types. As shown in Fig. 1A, at 37°C, procollagen I is present in a faint reticular pattern reminiscent of the endoplasmic reticulum (ER), which is the site of procollagen synthesis, with perinuclear enrichment that is likely to correspond to ER exit sites, the ER Golgi intermediate compartment (ERGIC) and the Golgi apparatus, key points along the secretory pathway. There was a slight increase in cellular procollagen I abundance in the CALFAN_1 cells (Fig. 1B). At 42°C, there was a striking increase in the abundance of procollagen I in cells from the two patients with CALFAN syndrome, which was predominantly in a reticular pattern reminiscent of the ER (Fig. 1A,B). Western blotting confirmed an increase in cellular procollagen I abundance in CALFAN cells compared to wild-type cells (Fig. 1C,D). A similar enrichment of cellular procollagen I in CALFAN cells was also observed in the absence of ascorbic acid, seen at both 37°C and an elevated temperature of 40°C (Fig. 1E). This increase was seen across fibroblasts derived from four different patients with CALFAN, and double labelling with the ER marker protein disulphide isomerase (PDI) confirmed that the bulk of procollagen I was present in the ER in the CALFAN cells (Fig. 1E). These results indicate that deficiency of SCYL1 lead to intracellular accumulation of procollagen I in the ER.

**Fig. 2.**
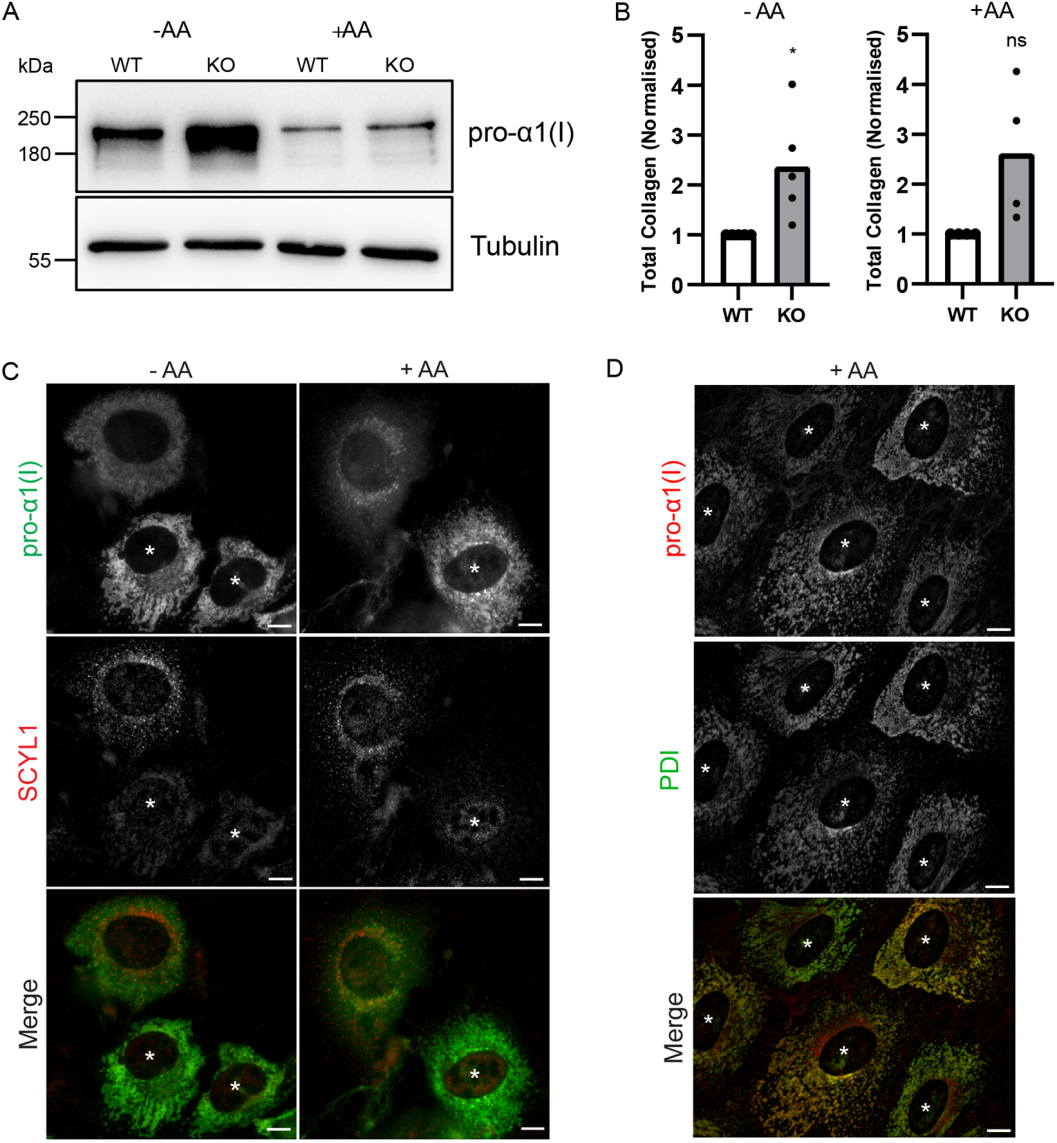
**Analysis of procollagen I abundance in SCYL1 knockout MEFs.** (A) Western blot of wild-type (WT) and SCYL1 knockout (KO) MEFs grown at 37°C for 2 days in the absence or presence of 0.25 mM ascorbic acid (-AA or +AA, respectively). Samples were blotted with antibodies against pro-α1(I) and tubulin; the latter was used as a loading control. (B) Total collagen was normalized to tubulin and WT values were set as 1. *n*=5 independent experiments for -AA and *n*=4 independent experiments for +AA. Unpaired *t*-test with Welch's correction, *P≤0.05, ns, not significant. (C) SCYL1 CRISPR KO MEFs were grown for three days in medium in the absence or presence of 0.25 mM AA. Following fixation cells were labelled with antibodies against procollagen pro-α1(I) (green) and SCYL1 (red). Asterisks denote SCYL1 KO MEFs. (D) SCYL1 CRISPR KO MEFs were grown for three days in medium supplemented with 0.25 mM AA. Following fixation cells were labelled with antibodies against procollagen pro-α1(I) (red) and PDI (green). Asterisks denote SCYL1 KO MEFs. Scale bars: 10 µm.

To further assess the effect of SCYL1 deficiency on procollagen I abundance, CRISPR-Cas9 was used to target *Scyl1* in immortalized mouse embryonic fibroblasts (MEFs) (Fig. S2A). We used three different single guide RNAs (sgRNA), hereafter referred to as G1, G2 and G3, to target the *Scyl1* locus, with most efficient loss of SCYL1 protein obtained using all three guides in combination (G4), as confirmed by western blotting and immunofluorescence microscopy (Fig. S2B,C). The SCYL1 KO MEFs were cultured in the absence or presence of ascorbic acid at 37°C and western blotting for procollagen I was performed. We could not analyze the MEFs at 42°C because neither wild-type nor SCYL1 KO cells were viable at this elevated temperature. Procollagen I was more abundant at steady-state in SCYL1 KO cells compared to wild-type control cells when cultured in the absence of ascorbic acid (Fig. 2A,B). As expected, in the presence of ascorbic acid, which promotes procollagen folding and export, intracellular levels of procollagen I were greatly reduced but, again, the cellular levels were higher in SCYL1 KO compared to wild-type cells (Fig. 2A,B). Immunofluorescence microscopy confirmed the increase in intracellular procollagen I in the SCYL1 KO MEFs (Fig. 2C). It also indicated procollagen I localization to a reticular network extending throughout the cytoplasm, which co-labeling confirmed corresponds to the ER (Fig. 2C,D). Thus, SCYL1 deficiency results in accumulation of procollagen I within the ER in KO fibroblasts.

### Procollagen I transcription and secretion kinetics are unaffected by SCYL1 deficiency

**Fig. 3.**
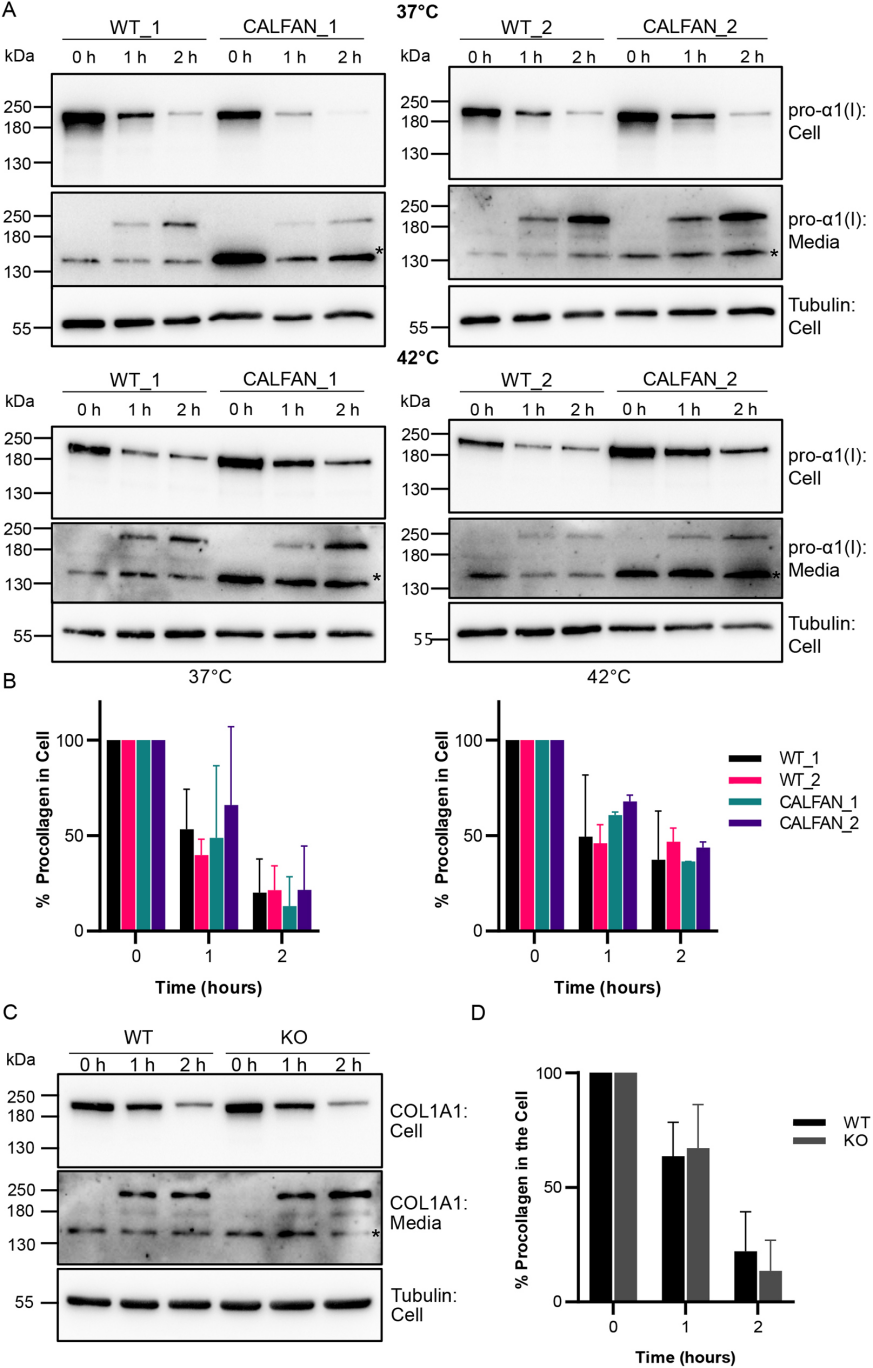
**Procollagen I trafficking in CALFAN fibroblasts and SCYL1 knockout MEFs.** (A) Western blots showing the analysis of procollagen I trafficking in different wild-type (WT; i.e. WT_1 or WT_2) and CALFAN fibroblasts (i.e. CALFAN_1 or CALFAN_2) fibroblasts. Cells were grown at 37°C (left panels) or 42°C (right panels) for 48 h prior to medium change and induction of traffic at either 37°C (top panel) or 42°C (bottom panel) in fresh medium containing ascorbic acid and cycloheximide. Medium and cell lysates obtained as indicated at 0, 2 or 2 h were blotted for pro-α1(I) and tubulin; the latter was used as a loading control. Asterisks indicate non-specific bands found in pro-α1(I) medium. (B) Bar graphs showing the abundance of procollagen quantified by normalizing cellular pro-α1(I) levels to those of tubulin and then expressing that as a percent of the equivalent value for time=0′ for each cell type. Error bars represent the mean +s.d.; *n*=2 independent experiments. Multiple unpaired *t*-tests with Welch's correction. Differences were found to be not significant. (C) Western blot analysis of procollagen I trafficking in wild-type (WT) and SCYL1 knockout (KO) MEFs. Cells were grown at 37°C for 48 h prior to medium change and induction of traffic in fresh medium containing ascorbic acid and cycloheximide. Medium and cell lysates at the indicated timepoints were blotted for pro-α1(I) and tubulin, which was used as a loading control. The asterisk indicates a non-specific band found in the pro-α1(I) medium blots. (D) Bar graph showing procollagen I abundance quantified by normalizing cellular pro-α1(I) levels to those of tubulin and then expressing that as a percent of the equivalent figure for time=0′ for both cell types. Error bars represent the mean +s.d.; *n*=4 independent experiments. Multiple unpaired *t*-tests with Welch's correction. Differences were found to be not significant.

To understand why SCYL1-deficient fibroblasts had increased amounts of procollagen I in the ER, we first examined its expression using qPCR. As shown in Fig. S3, there was no change in the expression of *COL1A1* in the CALFAN compared to wild-type fibroblasts, either at 37°C or 40°C, as assessed by *COL1A1* transcript levels. We next assessed trafficking of procollagen I, hypothesizing that ER accumulation of the protein is due to reduced ER export and trafficking along the secretory pathway. To assess procollagen I trafficking, we used an established pulse-chase method in which cells are cultured without ascorbic acid to allow ER accumulation of procollagen I, followed by a shift to medium containing ascorbic acid to promote ER export and secretory traffic in the presence of cycloheximide to block new protein synthesis (Mironov et al., 2001). As expected, we saw efficient trafficking of procollagen I into the medium in wild-type fibroblasts, with the majority exported following a 2-h chase period (Fig. 3A,B). Trafficking still occurred at 42°C, albeit with reduced efficiency, as indicated by greater cellular procollagen I levels at the 2-h timepoint (Fig. 3A,B) – which was expected according to a previous study (Bruckner and Eikenberry, 1984). Analysis of the CALFAN compared to control cells indicated that procollagen I export was unaffected at both 37°C and 42°C (Fig. 3A,B). To further assess procollagen I trafficking, we analyzed cells subjected to pulse-chase by immunofluorescence microscopy (Fig. S4A-C). Again, there was no significant difference in the rate of procollagen I trafficking between control and CALFAN cells, as assessed by analyzing transit to and through the Golgi apparatus, and subsequent export from the cell. Procollagen I trafficking was also studied in the SCYL1 KO MEFs, which – again – revealed no difference in the rate of secretory export when compared to that in wild-type MEFs (Fig. 3C,D). Together these data indicate that SCYL1 deficiency does not significantly affect procollagen I expression or secretory trafficking in fibroblasts.

### Altered ER morphology and increased ER stress upon SCYL1 deficiency

**Fig. 4.**
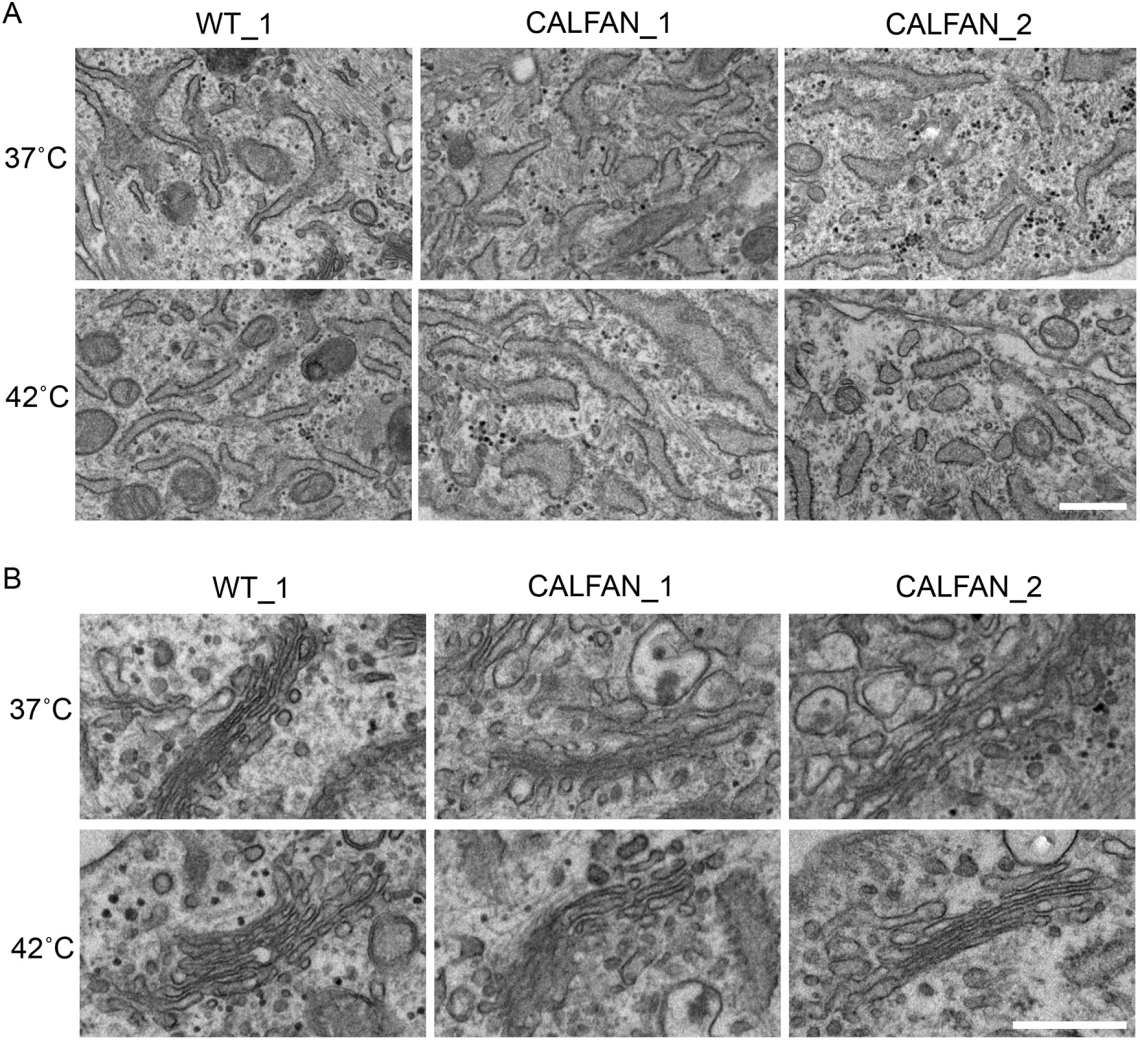
**Electron microscopy analysis of ER and Golgi in CALFAN fibroblasts.** (A) Transmission electron microscopy (TEM) images of the endoplasmic reticulum in wild-type (WT_1) fibroblasts and fibroblasts from patients with CALFAN (CALFAN_1 and CALFAN_2) cultured for 24 h at 37°C (top) or 42°C (bottom) in the presence of 0.25 mM ascorbic acid. (B) TEM images of the Golgi apparatus in WT fibroblasts and fibroblasts from patients with CALFAN cultured for 24 h at 37°C (top) or 42°C (bottom). Scale bars: 0.5 µm.

Accumulation of extracellular matrix proteins, such as procollagen in the ER lumen can result in swelling and distension of this compartment and is associated with the induction of ER stress (Boot-Handford and Briggs, 2010; Walter and Ron, 2011). We, therefore, wondered whether the increased abundance of procollagen I in the ER of cells from patients with CALFAN and in SCYL1 KO fibroblasts would lead to morphological changes in the ER, and whether an ER stress response would be induced. Immunofluorescence analysis revealed no gross changes in ER architecture in CALFAN compared to control fibroblasts, or in SCYL1 KO compared to wild-type MEFs (Fig. 1E and Fig. S4A,B). For higher resolution analysis of ER ultrastructure, electron microscopy was performed. This revealed a moderate dilation of ER cisternae in the CALFAN fibroblasts compared to control cells at 37°C, with a greater effect seen upon culturing cells at 42°C (Fig. 4A). Previous work has shown that RNAi-mediated depletion of SCYL1 causes expansion of the Golgi apparatus and dilation of cisternae in HeLa cells (Burman et al., 2010). We, therefore, also analyzed Golgi morphology in the CALFAN cells by using electron microscopy. Fibroblasts from both patients with CALFAN had a morphologically normal Golgi apparatus, with clearly stacked cisternae and no obvious distension of the cisternae themselves (Fig. 4B). This morphology was observed at 37°C and 42°C, indicating that loss of SCYL1 in CALFAN fibroblasts while causing ER dilation, does not affect Golgi morphology.

**Fig. 5.**
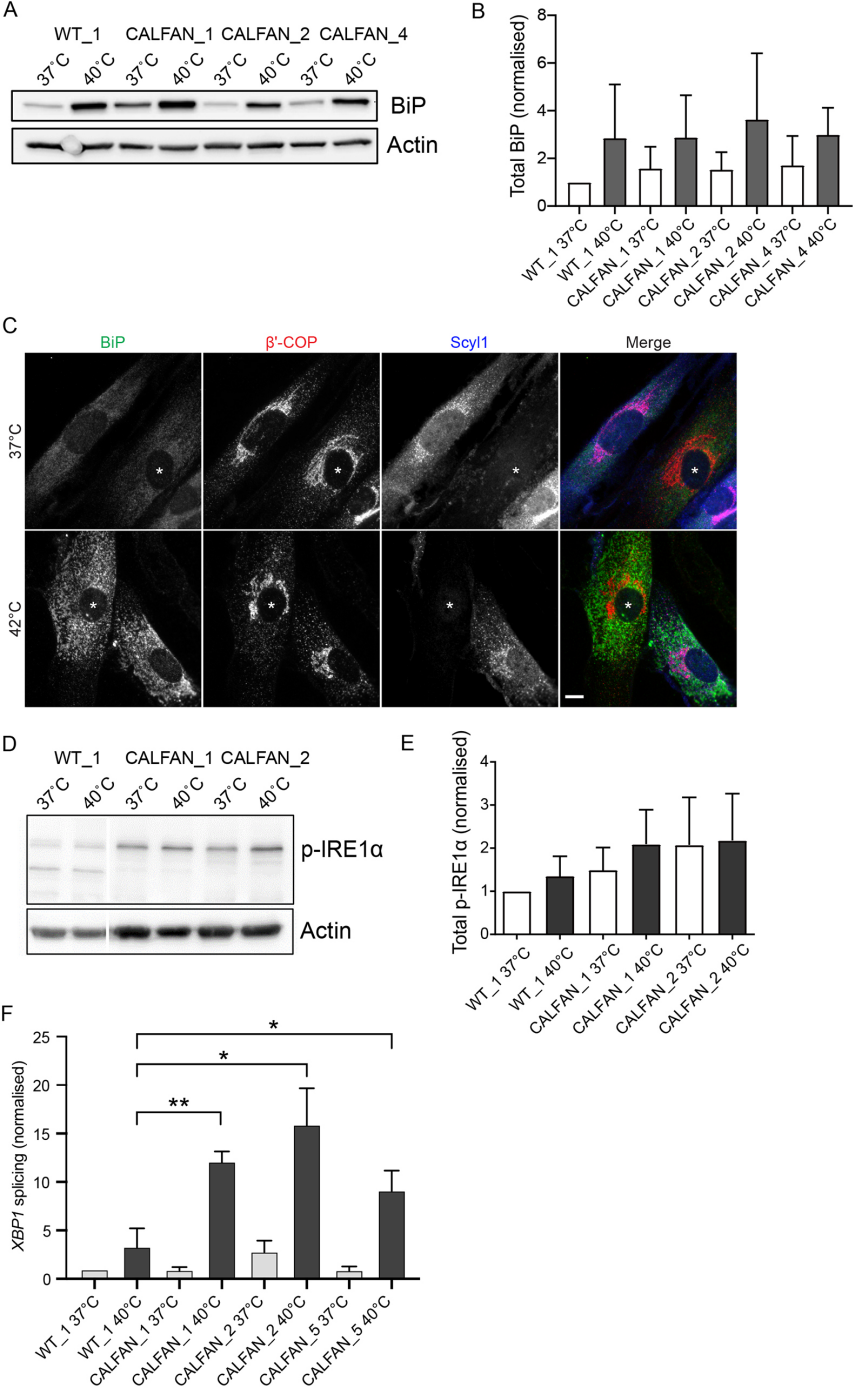
**Analysis of ER stress in CALFAN fibroblasts.** (A,B) Western blot of wild-type (WT_1) and CALFAN fibroblasts (CALFAN_1, CALFAN_2, CALFAN_4) that were grown separately for two days at 37°C in culture medium lacking exogenous ascorbic acid and then, for the final 24 h, were either kept at 37°C or at 40°C. Lysates were incubated with antibodies against BiP and actin; the latter was used as loading control. (A). (B) Bar graph showing total BiP levels of cells as described for A, quantified relative to actin levels and normalized to levels of WT_1 at 37°C. Error bars represent mean +s.d.; *n*=3 independent experiments. Multiple unpaired *t*-tests with Welch's correction. Differences were found to be not significant. (C) Wild-type (WT) and CALFAN fibroblasts were grown together as indicated for two days in medium containing 0.25 mM ascorbic acid. For the final 24 h cells were either kept at 37°C or shifted to 42°C, prior to fixation and labelling with antibodies against BiP (green), the COPI subunit β′-COP (red), SCYL1 (blue). CALFAN fibroblasts are denoted by asterisks. Scale bar: 10 µm. (D) Western blot of cells cultured as described for A; antibodies used were against phosphorylated-IRE1α and actin; the latter was used as loading control. (E) Levels of phosphorylated IRE1α (p-IRE1α) form cells as indicated were quantified relative to actin and normalized to WT_1 cultured at 37°C. Error bars represent mean +s.d.; *n*=3 independent experiments. Multiple unpaired *t*-tests with Welch's correction. Differences were found to be not significant. (F) Bar graph showing levels of *XBP1* splicing in cells cultured as described for A. RNA was isolated, reverse transcribed and spliced *XBP1* mRNA measured by qPCR. Error bars represent the mean +s.d.; *n*=3 independent experiments. Multiple unpaired *t*-tests with Welch's correction. Differences were found to be significant in CALFAN cells at 40°C (*P<0.05 vs control, **P<0.01)**.**

We next analyzed whether ER stress was increased in the CALFAN fibroblasts compared to controls. Cells were incubated at 37°C or an elevated temperature of 40°C, and several ER stress markers were analyzed. This was done in the absence of exogenous ascorbic acid because pilot experiments did not reveal a difference in the ER stress phenotype. We observed an increase in the abundance of the ER chaperone BiP, whose expression is stimulated by ER stress signaling, when cells were cultured at elevated temperature, assessed by western blotting (Fig. 5A,B) and immunofluorescence microscopy (Fig. 5C). However, in both cases BiP levels were similar in control and CALFAN cells, seen at 37°C and at elevated temperatures of 40°C or 42°C, where BiP expression is elevated compared to 37°C (Fig. 5A-C). Analysis of the three main ER stress sensor pathways revealed a trend towards increased levels of phosphorylated ERN1 (hereafter referred to as IRE1α) in CALFAN compared to control cells, most apparent at 40°C (Fig. 5D,E). IRE1α phosphorylation lies upstream of mRNA splicing of *XBP1*, a gene encoding an ER stress-dependent transcription factor that is a sensitive measure of ER stress levels (Walter and Ron, 2011). RT-PCR was used to assess splicing of *XBP1*, which was significantly increased in CALFAN cells compared to control cells at the elevated temperature of 40°C. As expected, *XBP1* splicing was increased upon the shift of temperature from 37°C to 40°C in control cells, consistent with increased ER stress; however, the magnitude of increase was significantly greater in the CALFAN fibroblasts (Fig. 5F). Analysis of the other two major ER stress response pathways − one mediated by ATF6, during which cleavage to the 50 kDa form occurs upon ER stress, and the other mediated by PERK that is phosphorylated in response to ER stress – revealed no significant differences between the cells from patients with CALFAN and control cells (Fig. S5A-C). Thus, SCYL1 deficiency in CALFAN fibroblasts leads to increased ER stress at elevated temperature, shown most clearly by using *XBP1* splice levels as a readout.

### Deficiency of SCYL1 causes increased cell death at elevated temperature

**Fig. 6.**
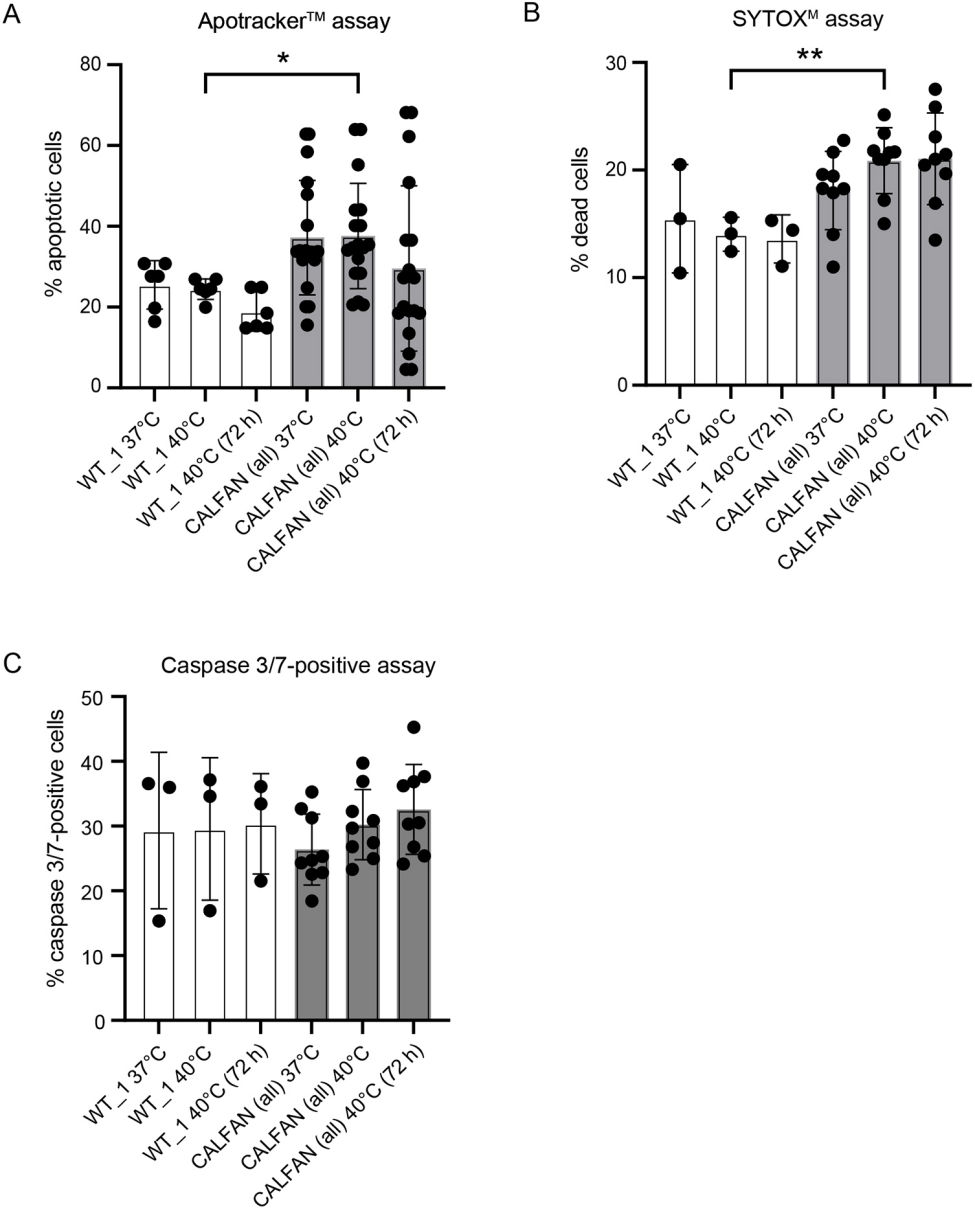
**Apoptosis and cell death of CALFAN fibroblasts analyzed using flow cytometry.** (A) Apoptotic cells were calculated as the percentage of apoptotic cells (Apotracker™-positive) relative to the total number of cells analyzed. Cells were maintained at 37°C or shifted to 40°C for 48 h, or 72 h as indicated. (B) Dead cells were calculated as the percentage of SYTOX™-positive cells relative to the total number of cells, at 37°C and after shift to 40°C for 48 h, or 72 h as indicated. (C) Caspase 3- and caspase 7-positive (caspase 3/7-positive) cells were calculated as the percentage of caspase 3/7-positive cells (CellEvent™ Caspase 3/7) relative to the total number of cells at 37°C and after shift to 40°C for 48 h or 72 h as indicated. CALFAN (all) indicates data averaged across CALFAN_1, CALFAN_2 and CALFAN_5 cells. Data are expressed as the mean±s.d. of three experiments. Multiple unpaired *t*-tests with Welch's correction (*P<0.05, **P<0.01).

Unresolved ER stress can cause cell death by apoptosis. We, therefore, analyzed levels of apoptosis in SCYL1-deficient fibroblasts, both at 37°C or following a shift to elevated temperature of 40°C for 48 h or 72 h. We used several assays to measure apoptosis and cell death. Translocation of phosphatidylserine to the external leaflet of the cellular membrane, indicating loss of membrane asymmetry during apoptosis, was assessed using the Apotracker^TM^ assay, indicating increased apoptosis in CALFAN cells compared to wild-type control cells (Fig. 6A). Increased apoptosis was observed at both 37°C and 40°C, and did not increase upon prolonged incubation at 40°C (Fig. 6A). Cell death was assessed using SYTOX^TM^ dye, which binds to DNA upon loss of cell membrane integrity. Cell death was increased in the CALFAN cells at both 37°C and 40°C, with a trend to increased death at 40°C, but without further increase upon prolonged incubation at 40°C (Fig. 6B). Interestingly, analysis of levels of active caspases 3 and 7, did not indicate increased activity in CALFAN cells compared to wild-type control cells (Fig. 6C), suggesting that the observed increased levels of cell death in CALFAN cells is caspase-independent.

## DISCUSSION

ER stress is associated with numerous disease types, including infectious disease, rare genetic conditions and more common diseases, such as cancer, diabetes and Alzheimer's disease (Wang and Kaufman, 2016). All tissues can be affected, including the nervous system, liver and skeletal system, all of which are relevant in the context of CALFAN syndrome (Briggs et al., 2020; Hetz and Saxena, 2017; Liu and Green, 2019). A previous study by Lenz and colleagues did not report an ER stress response in cells from patients with CALFAN (Lenz et al., 2018); however, no systematic examination of ER stress pathway signaling had been carried out in that study, and increased – febrile – temperature had not been investigated (Lenz et al., 2018). In our current study, we observed that the IRE1α/XBP1 arm of ER stress signaling is activated in CALFAN fibroblasts and, importantly, that the effect was more pronounced at elevated temperatures. It is known that an increase in temperature induces ER stress, which – if unresolved – can cause cell death (Sano and Reed, 2013). We showed here that the CALFAN cells are more susceptible to this effect. CALFAN cells also underwent more apoptosis and cell death than wild-type cells, with a trend to increased death at elevated temperatures. Interestingly, activity of caspase 3 and caspase 7 appeared unaltered in the CALFAN cells, suggesting that cell death is occurring in a caspase-independent manner. Further studies will be required to determine the precise mechanism by which CALFAN cells undergo cell death. Nevertheless, increased cell death may account for the induction of liver pathology seen in patients with CALFAN syndrome in the febrile state. Interestingly, in cells derived from patients with mutations in NBAS, which shares similar liver pathology to CALFAN syndrome that can also be induced by fever, gene expression changes indicative of ER stress induction were observed (Chavany et al., 2020; Haack et al., 2015). Thus, ER stress might underlie the liver pathology of both CALFAN syndrome and NBAS deficiency. We also think that ER stress might underlie the neurological and skeletal phenotypes of CALFAN syndrome and, possibly, those seen in NBAS deficiency, considering the strong association of ER stress with neurodegeneration and skeletal abnormalities. We observed ER stress in fibroblasts, indicating that this phenomenon occurs in extracellular matrix-producing cells, consistent with effects on the matrix-rich tissues of the skeletal system in patients with CALFAN. Moreover, SCYL1 KO mice, which recapitulate the neurological phenotype of CALFAN syndrome (Pelletier et al., 2012; Schmidt et al., 2007), show prominent aggregation of TDP-43 (also known as TARDBP) within their motor neurons. While the cause of this aggregation, which is common to various neurodegenerative conditions, remains to be determined, it is interesting to note a strong functional association of TDP-43 aggregation with ER stress (de Mena et al., 2021), which may also be occurring in CALFAN syndrome.

We observed accumulation of the major secreted protein procollagen I in the ER in SCYL1-deficient cells, which was more pronounced at elevated temperatures, correlating with the increased ER stress induction and cell death at these temperatures. Because it is known that accumulation of misfolded proteins in the ER triggers ER stress (Walter and Ron, 2011), and that protein misfolding is increased at higher temperatures, we hypothesize that accumulation of secretory cargo in the ER is likely to account for the ER stress induction we observed in SCYL1-deficient cells. What is less clear is the cause of procollagen I accumulation in the ER. Neither procollagen I expression, assessed at mRNA levels, nor defective protein trafficking from the ER seem to be the underlying cause. However, it remains possible that a slight kinetic delay in procollagen I transport out of the ER is occurring, such that our pulse-chase experiments failed to detect significant differences. More sensitive trafficking assays may help in this regard. It is also possible that the ER export of cargo proteins other than procollagen I is sensitive to loss of SCYL1, causing these proteins to accumulate in the ER and trigger ER stress. Another possibility is that folding or turnover of cargo in the ER lumen is compromised in the absence of SCYL1. Since SCYL1 is required for efficient Golgi to ER retrograde transport (Burman et al., 2008; Lenz et al., 2018), it is possible that cycling of chaperones or factors required for ERAD from the Golgi back to the ER is compromised in the SCYL1-deficient cells. Interestingly, upon induction of ER stress ATF6 is trafficked from the ER to the Golgi where it undergoes cleavage and activation (Walter and Ron, 2011). We observed ATF6 cleavage in the CALFAN cells similar to that in the controls, indicating that ER exit of this important stress signaling protein is not inhibited upon SCYL1 deficiency. ATF6 does not cycle from the Golgi back to the ER (Shen et al., 2002) and, so, any defects in retrograde transport would not be expected to alter ATF6 dynamics and cleavage.

This study suggests a role for ER stress in CALFAN syndrome pathology. However, it remains possible that other mechanisms exist alongside ER stress induction or that ER stress induction leads to downstream cellular defects that compound the phenotype. Accumulation of misfolded proteins in the ER and induction of ER stress can lead to an increase in ER-phagy, whereby the ER is digested by incorporation into autophagosomes that subsequently fuse with lysosomes for degradation (Bernales et al., 2007). A recent study has shown that loss of SCYL1 can disrupt the positioning and morphology of lysosomes (Kaeser-Pebernard et al., 2022), suggesting an involvement in lysosome homeostasis and, by extension, autophagic clearance of substrates. Kaeser-Pebernard and colleagues also have shown changes in exosome release in SCYL1 KO cells (Kaeser-Pebernard et al., 2022). We have observed an increase in lysosome abundance in fibroblasts of patients with CALFAN (data not shown), suggesting a role in lysosome homeostasis that could impact upon downstream processes, such as autophagy. This is of interest, as recessive ataxias often show clinical overlap with lysosomal disorders, and because many lysosomal diseases (e.g. Tay-Sachs disease) show evidence of clinical features of ataxia and a Purkinje cell loss (Platt et al., 2018; Synofzik and Nemeth, 2018).

Interestingly, CALFAN arises from hypomorphic mutations in SCYL1, with residual expression detected in those cases where levels of the protein were analyzed (Incecik et al., 2018; McNiven et al., 2021; Schmidt et al., 2015; Youssef et al., 2023; Chavany et al., 2020; Isa et al., 2023; Li et al., 2019; Shohet et al., 2019; Smith et al., 2017; Spagnoli et al., 2019; Yigit et al., 2022; Lenz et al., 2018). This suggests that complete loss of SCYL1 is incompatible with life. However, SCYL1 KO mice are viable (Pelletier et al., 2012; Schmidt et al., 2007), and double KO of SCYL1 and its paralogue SCYL3, which is also Golgi-localized and binds COPI, also generates viable mice, albeit with an accelerated neurodegenerative phenotype (Kuliyev et al., 2018). This would – at least in mice – argue against redundancy between SCYL1 and SCYL3. However, it remains possible that SCYL3 can partially functionally substitute for loss of SCYL1 in humans. It is also possible that other compensatory or adaptive mechanisms come into play in the absence of SCYL1, which may alleviate some of the cellular deficits seen upon SCYL1 deficiency. Further work will be required to fully characterize CALFAN syndrome etiology. It will be interesting in the future to determine the global changes that occur upon SCYL1 deficiency in CALFAN syndrome at the level of gene expression, proteostasis, and in terms of secretory trafficking and ER stress induction.

## MATERIALS AND METHODS

### Antibodies

The following antibodies were used in this study: mouse anti-β′-COP [COPB2] [Rainer Pepperkok, EMBL Heidelberg, Germany; 1:100 for immunofluorescence (IF)]; rabbit anti-collagen [type I collagen alpha-1 amino-propeptide (Kerafast, LF-68, 1:100 for IF and 1:1000 for western blotting (WB)]; mouse anti-GAPDH (Santa Cruz Biotechnology, sc-365062, 1:4000 for WB); mouse anti-GM130 (BD Biosciences, 610823, 1:100 for IF); rabbit anti-GM130 (available on request from M.L.; 1:100 for IF); rabbit anti-GRP78 (BiP) (Abcam, ab21685, 1:200 for IF) and rabbit anti-GRP78 (BiP) (Cell Signaling, 3177, 1:1000 for WB); mouse anti-PDI (Enzo Life Sciences, ADI-SPA-891-F, 1:200 for IF) and rabbit anti-PDI (Cell Signaling, 35016, 1:200 for IF, 1:1000 for WB); sheep anti-SCYL1 (available on request from M.L.; 1:100 for IF and 1:1000 for WB) and rabbit anti-SCYL1 (Sigma-Aldrich, HPA015015, 1:100 for IF and 1:1000 for WB); rabbit anti-IRE1α (Cell Signaling, 3294; 1:1000 for WB); rabbit anti-phosphorylated IRE1α (Invitrogen, PA1-16927, 1:1000 for WB); rabbit anti-PERK (Cell Signaling, 5683; 1:1000 for WB); rabbit anti-phosphorylated PERK (Invitrogen, PA5-40294; 1:1000 for WB); anti-ATF6 (Santa Cruz, SC-166659; 1:1000 for WB) mouse anti-β-actin conjugated to horseradish peroxidase (anti-β-actin-HRP; Santa Cruz, SC47778, 1:1000 for WB); mouse anti-tubulin (provided by Keith Gull, University of Oxford, UK or Wanjin Hong, Institute of Molecular and Cell Biology, Singapore; both used at 1:1000 for WB). HRP-conjugated secondary antibodies for WB were from Sigma-Aldrich (A4416 and A0545, both used at 1:1000) or Santa Cruz Biotechnology (sc-2354, 1:1000). Antibodies conjugated to Alexa Fluor-488, 555, 594 or 647 were from Jackson ImmunoResearch Laboratories or Invitrogen.

### Cell culture

Cells were grown at 37°C under 5% CO_2_, except for experiments that required growth at 40°C or 42°C under 5% CO_2_. Cells of the Lenti-X 293 T transformed HEK 293 cell line (Takara Bio) were cultured in Dulbecco's modified Eagle's medium (DMEM) high glucose (Sigma-Aldrich) supplemented with 10% (v/v) fetal bovine serum (FBS; Thermo Fisher Scientific), 1% (v/v) penicillin-streptomycin solution (Sigma-Aldrich) and 4 mM glutamine (Sigma-Aldrich). Human skin fibroblasts were cultured in DMEM high glucose supplemented with 20% (v/v) FBS, 1% (v/v) penicillin-streptomycin solution and 1% (v/v) non-essential amino acids (Biological Industries or Cytivia). Informed consent was obtained from all patients or from their parents in the case of patients under the age of 18 years. The study was approved by the ethical committee of the Technische Universität München and the ethical committee of the University Hospital Heidelberg. MEFs (ATCC) were cultured in DMEM+GlutaMAX medium (Thermo Fisher Scientific) supplemented with 10% (v/v) FBS and 1% (v/v) penicillin-streptomycin solution. PBS and trypsin-EDTA solution were purchased from Sigma-Aldrich. For experiments where ascorbic acid (L-ascorbic acid 2-phosphate sesquimagnesium salt hydrate; Sigma-Aldrich) was in the medium, this was added at a concentration 0.25 mM (1:2000 dilution from 40 mg/ml stock).

### Generation of SCYL1 knockout MEFs using CRISPR-Cas9

*Scyl1* sgRNA CRISPR/Cas9 All-in-One Lentivector set was purchased from Applied Biological Materials Inc. (#432161140595) and contained three single guide RNAs (sgRNA). Lenti-X 293T cells were seeded in 10 cm dishes to be 80% confluent after 24 h. Cells were co-transfected with 5 µg *SCYL1* sgRNA (or 2 µg of all three *SCYL1* sgRNA), 3 µg psPAX2 vector and 2 µg pMD2 vector (lentiviral packaging vectors). This was diluted in 900 µl of Opti-MEM, to which 20 µl FuGENE HD (Promega) was added, before incubation at room temperature for 8 min. Following incubation, the mixture was added directly to the cells. After 24 h the medium was replaced. After a further 24 h medium was collected and stored at 4°C. Fresh medium was added to the cells and collected after a further 24 h before being pooled with the previously collected medium. Pooled medium was filtered through a 0.45 µm filter (EMD Millipore). Produced viruses were either used directly or snap frozen and stored at −80°C. MEFs were seeded in T25 flasks to be 80–90% confluent at the time of transduction. Lentivirus solution (2.5 ml) was mixed with fresh medium at a 1:1 ratio and added to the cells. After 24 h the medium was replaced with new one. After another 24 h, puromycin (Sigma-Aldrich) at a final concentration of 2 µg/ml, was added to the cells. Medium containing puromycin was replaced daily until all non-transfected control cells had died. Cells were passaged twice before use in experiments. Efficiency of SCYL1 knockout (KO) was assessed by western blotting and immunofluorescence microscopy.

### Procollagen I trafficking experiments

For immunofluorescence analysis cells were seeded according to their respective growth rates into 6-well plates containing glass coverslips in the wells. After 24 h, plates were either kept at 37°C or moved to an incubator at 42°C for a further 48 h. Medium was removed and replaced with fresh medium containing 100 µg/ml cycloheximide (Sigma-Aldrich) and 0.25 mM L-ascorbic acid (Sigma-Aldrich). At times indicated, cells were washed twice with PBS before fixation with 3% PFA for 20 min at room temperature. For western blot analyses the protocol used was as described by Lekszas et al. (2020). Cells were seeded according to their respective growth rates into 6-well plates and grown at 37°C for 24 h, at which point plates were either kept at 37°C or moved to an incubator at 42°C for a further 48 h. Medium was removed and replaced with fresh medium containing 50 µM cycloheximide and 0.25 mM ascorbic acid. Cell lysates and medium were collected at indicated time points, and analyzed by SDS-PAGE and western blotting.

### Preparation of cell lysates and western blotting

Cells were washed twice with ice cold PBS and then lysed in HMNT buffer (20 mM HEPES pH 7.4, 0.1 M NaCl, 5 mM MgCl_2_, 0.5% Triton X-100) or RIPA buffer (150 mM NaCl, 50 mM Tris-HCl pH 8, 5 mM EDTA, 1% Nonidet P-40, 0.1% SDS, and 0.5% Na-deoxycholate) supplemented with protease inhibitor cocktail (Roche). Lysates were incubated on a shaking platform for 15−20 min at 4°C, before centrifugation at 13,200 rpm for 15 min at 4°C. Protein concentration was measured and cell extracts were snap frozen and stored at −80°C. Prior to running lysates an appropriate volume of 2× or 5× SDS sample buffer was added and samples were heated at 95°C for 5−10 min. Following separation by SDS-PAGE, proteins were transferred onto Amersham Protran nitrocellulose membrane (Cytivia). Following transfer membranes were blocked by incubation in blocking buffer [5% (w/v) dried skimmed milk (Marvel or Bio-Rad) in PBS containing 0.1% Tween-20 (PBST)] for 45 min at room temperature. Membranes were then incubated with primary antibody (diluted in blocking buffer) at 4°C overnight. Membranes were washed with PBST before incubation with HRP-conjugated secondary antibody (diluted in blocking buffer) for 1 h at room temperature. Protein bands were visualized by using ECL chemiluminescent substrate SuperSignal West Pico (Thermo Fisher Scientific), SuperSignal West Femto maximum sensitivity (Thermo Fisher Scientific), Pierce ECL western blotting substrate (Thermo Fisher Scientific) or Forte western HRP substrate (EMD Millipore) on a ChemiDoc (Bio-Rad) or Fusion (Vilber Lourmat) imaging system.

### Immunofluorescence microscopy

Cells were grown on glass coverslips and washed twice with PBS prior to fixation in 3% paraformaldehyde (PFA, Sigma-Aldrich) in PBS for 20 min at room temperature. Cells were then washed three times with PBS, with the second wash including 10 mM glycine pH 8.5 quench. Cells were permeabilized with 0.1% Triton X-100 in PBS (v/v) for 4 min at room temperature and then washed a further three times with PBS. Cells were incubated with primary antibody solution for 1 h at room temperature (1.5 h for MEFs), before three washes with PBS. Coverslips were then incubated with secondary antibody solution for 1 h at room temperature, before three washes with PBS. Coverslips were then mounted in Antifade mounting medium with DAPI (Vectashield or Invitrogen). Prepared slides were analyzed using either an Olympus BX60 upright microscope (60x/1.4 numerical aperture oil immersion objective) equipped with a CoolSNAP EZ camera (Photometrics) driven by MetaVue software (Molecular Devices), a Leica DMI4000 B or on a Zeiss Axio Imager Z2 LSM800 confocal microscope in 2D mode. Images were processed using ImageJ/Fiji software (NIH).

### Flow cytometry

Flow cytometry analyses were employed using staining against phosphatidylserine (Apotracker™), caspase-3 and caspase-7 as well as SYTOX™ AADvanced™ staining dead cells. Fibroblast cells were cultured for 48 h and incubated for 72 h at 37°C or 40°C, followed by collection in 15 ml centrifuge tubes. The cell pellets were resuspended in 500 µl BSA (0.5%) in PBS and transferred to 1.5 ml tubes, followed by staining with Apotracker (Apotracker™ Tetra Alexa Fluor^®^ 647; Biolegend) together with caspase-3 and caspase-7 (CellEvent™ Caspase-3/7 Green Flow Cytometry Assay Kit, Thermo Fisher Scientific) and Sytox AADvanced (SYTOX™ AADvanced™ Blue Dead Cell Stain Kit, Thermo Fisher Scientific), respectively. Of the Caspase-3/7 Green Detection Reagent, 0.5 µl was added to the tubes; samples were incubated for 15 min at 37°C, followed by addition of 5 µl Apotracker™ tetramer solution. Samples were incubated for a further 15 min at 37°C. In the last 5 min of incubation, 0.5 µl of the 1 mM SYTOX™ AADvanced™ Dead Cell Stain in DMSO was added. After transfer to FACS Analyser Tubes the samples were immediately analyzed using a flow cytometer (Beckman Coulter). Gates were set according to unstained control samples, and the analyses were performed using Kaluza Analysis Software (Beckman Coulter).

### RT-qPCR

Total RNA was extracted from cells using the peqGOLD RNA Kit (VWR International GmbH, Darmstadt, Germany) following the manufacturer's instructions. Isolated RNA was subjected to DNase treatment (pegGOLD DNase Kit) to remove contaminating DNA. 1 µg of resulting purified RNA was used to prepare cDNA with the help of SuperScript III Reverse Transcriptase (Invitrogen, Germany). For expression analysis using reverse transcription quantitative PCR (RT-qPCR), we used PerfeCTa® SYBR^®^FastMix™ (VWR, Germany) with template cDNA and primer concentration according to the manufacturer's protocol. Primer sequences were: *XBP1* – forward: 5′-GGTCTGCTGAGTCCGCAGCAGG-3′; reverse: 5′-GGGCTTGGTATATATGTGG-3′; fragment length: 311 bp; *COL1A1* – forward: 5′-CGATGGATTCCAGTTCGAGTA-3′; reverse: 5′-GTTTACAGGAAGCAGACAGG-3′; length 400 bp. All primers had previously been verified for specificity by gel electrophoresis. PCRs were run on Bio-Rad CFX Connect RT-PCR Detection system at a preset melting cycle, with annealing temperature specific for each primer set. For quantification, actin RNA expression was used as an endogenous reference. Expression data were quantified using the 2−ΔΔCT method and are stated as fold-change in gene expression for each individual gene.

### Electron microscopy

MEF cells were grown on a 10-cm dish until confluency. Cell samples were fixed by adding double concentrated fixative directly to the same amount of medium in the culture dish, so that the final fixative was 4% formaldehyde+2.5% glutaraldehyde in 0.1 M HEPES buffer pH 7.2. Then samples were post-fixed with reduced osmium (1% osmium tetroxide+1.5% potassium ferrocyanide) in 0.1 M cacodylate buffer (pH 7.2) for 1 h, then in 1% uranyl acetate in H_2_O overnight. The samples were dehydrated in ethanol series infiltrated with TAAB LV resin and polymerized for 24 h at 60°C. Sections were cut with a Reichert Ultracut ultramicrotome and observed with a FEI Tecnai 12 Biotwin microscope at 100 kV accelerating voltage. Images were taken with a Gatan Orius SC1000 CCD camera.

### Statistics

Statistical analyses were conducted using GraphPad Prism software (GraphPad Software, San Diego, CA). A D'Agostino-Pearson normality test (omnibus K2) was conducted to assess whether data had a normal distribution. Where data did not have a normal distribution a ROUT outlier test (Q=1%) was carried out. Depending on the data and the result of the normality test either an unpaired *t*-test or Mann–Whitney test was performed, as indicated in figure legends.

### Reagent availability

Reagents used in this study can be obtained by contacting the corresponding authors.

## Supplementary Material

10.1242/dmm.052371_sup1 Supplementary information
