# Supplementary material for: SCYL1 deficiency in CALFAN syndrome is associated with ER stress and cell death
Source: Dis Model Mech. 2025 Nov 27;18(11):dmm052371. doi: 10.1242/dmm.052371 (PMC12690549; doi:10.1242/dmm.052371)
Supplement: Supplementary information [file dmm-18-052371-s1.pdf]

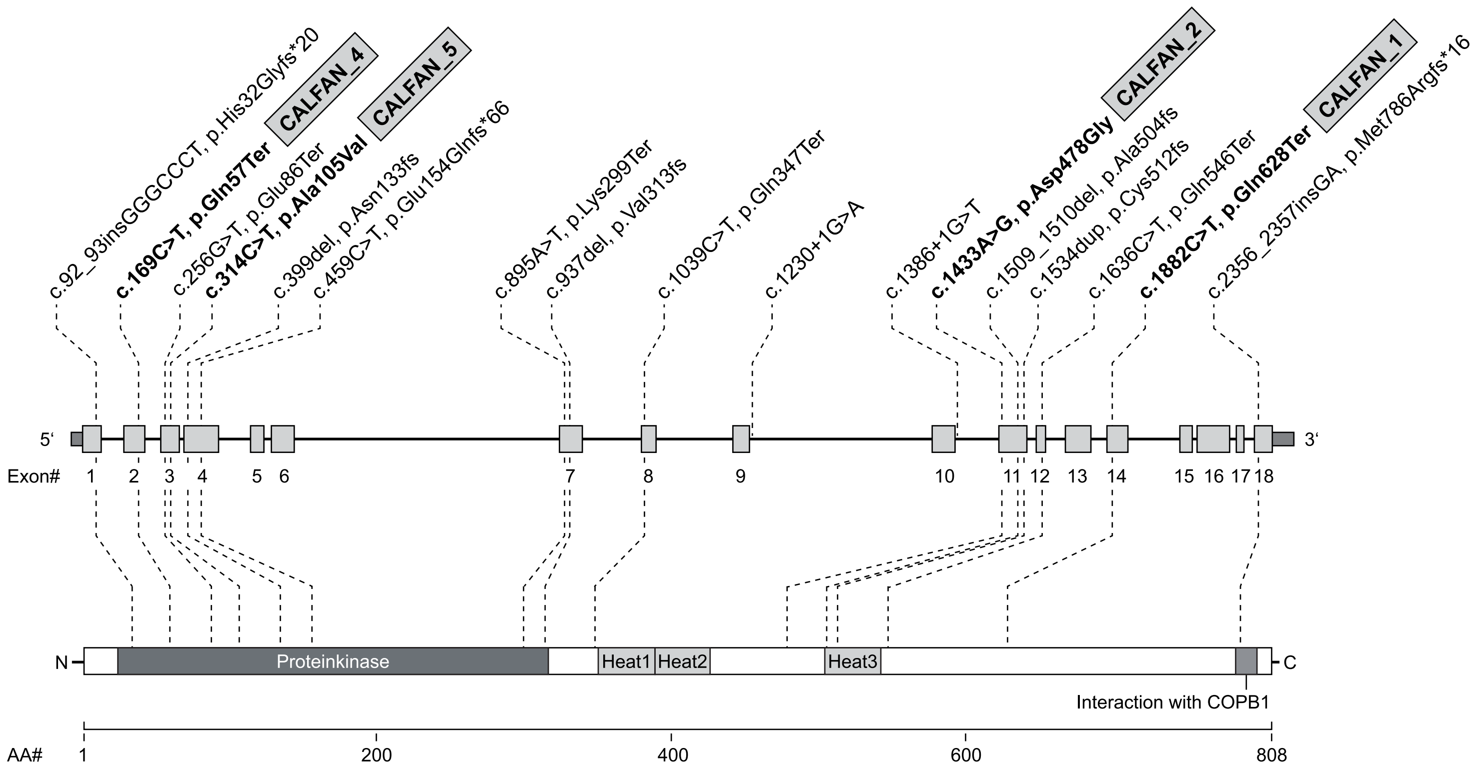

Fig. S1. LocalizationofSCYL1variants. All knownpathogenic variants includingaffected regionof SCYL1 proteinare shown. Variants present in ourpatients are displayed inbold.

Disease Models & Mechanisms • Supplementary information

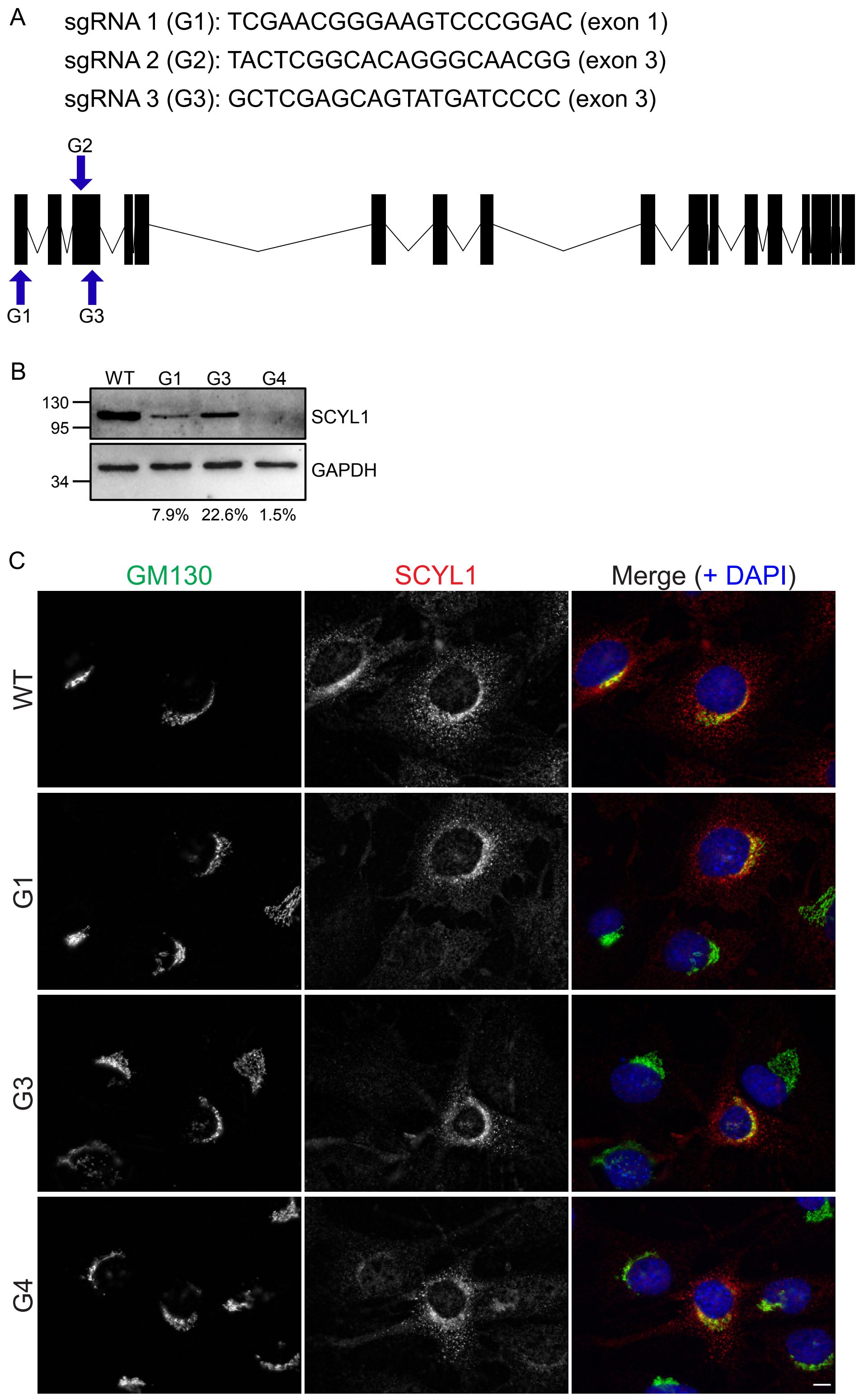

Fig. S2. Generation of CRISPR-Cas9-generated SCYL1 knockout MEFs. A. Sequences of the sgRNAs used to knockout SCYL1 from MEFs in CRISPR-Cas9 and the exon-intron structure of the SCYL1 gene in mice. Locations targeted by the gRNAs are indicated by blue arrows. **B)** Western blot analysis of SCYL1 in MEFs infected with lentivirus containing sgRNA 1 or 3 (G1 or G3) or containing a mix of all three sgRNA (G4). Percentage figures indicate SCYL1 protein levels compared to wild-type. GAPDH was used as a loading control. **C)** Wild-type and SCYL1 CRISPR KO MEFS as in B were grown for three days before fixation and labelling with antibodies against GM130 (green) and SCYL1 (red). Scale bar, 10 µm.

Disease Models & Mechanisms • Supplementary information

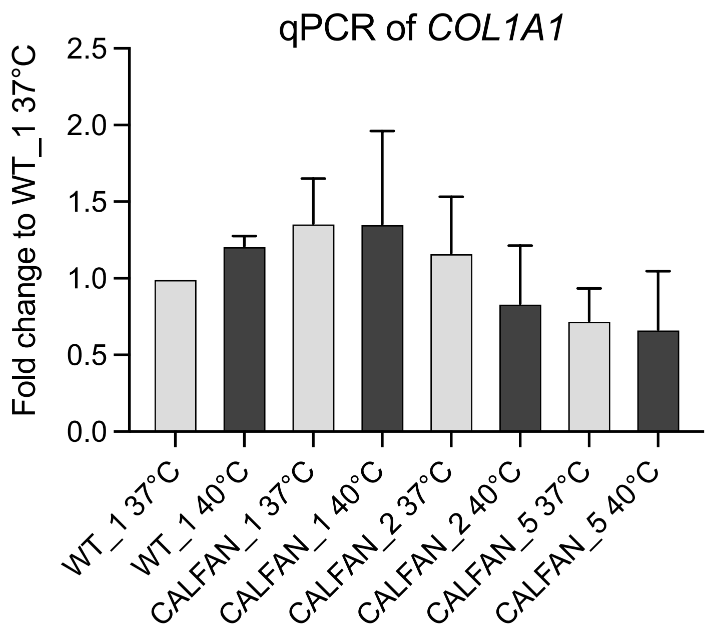

Fig. S3. QPCR of COL1A1. Wild-type and CALFAN fibroblasts were grown separately for two days in media. For the final 24 hours cells were either kept at 37°C or moved to an incubator at 40°C. RNA was isolated, reverse transcribed and COL1A1 amplified. No significant differences were noted on a transcriptional level.

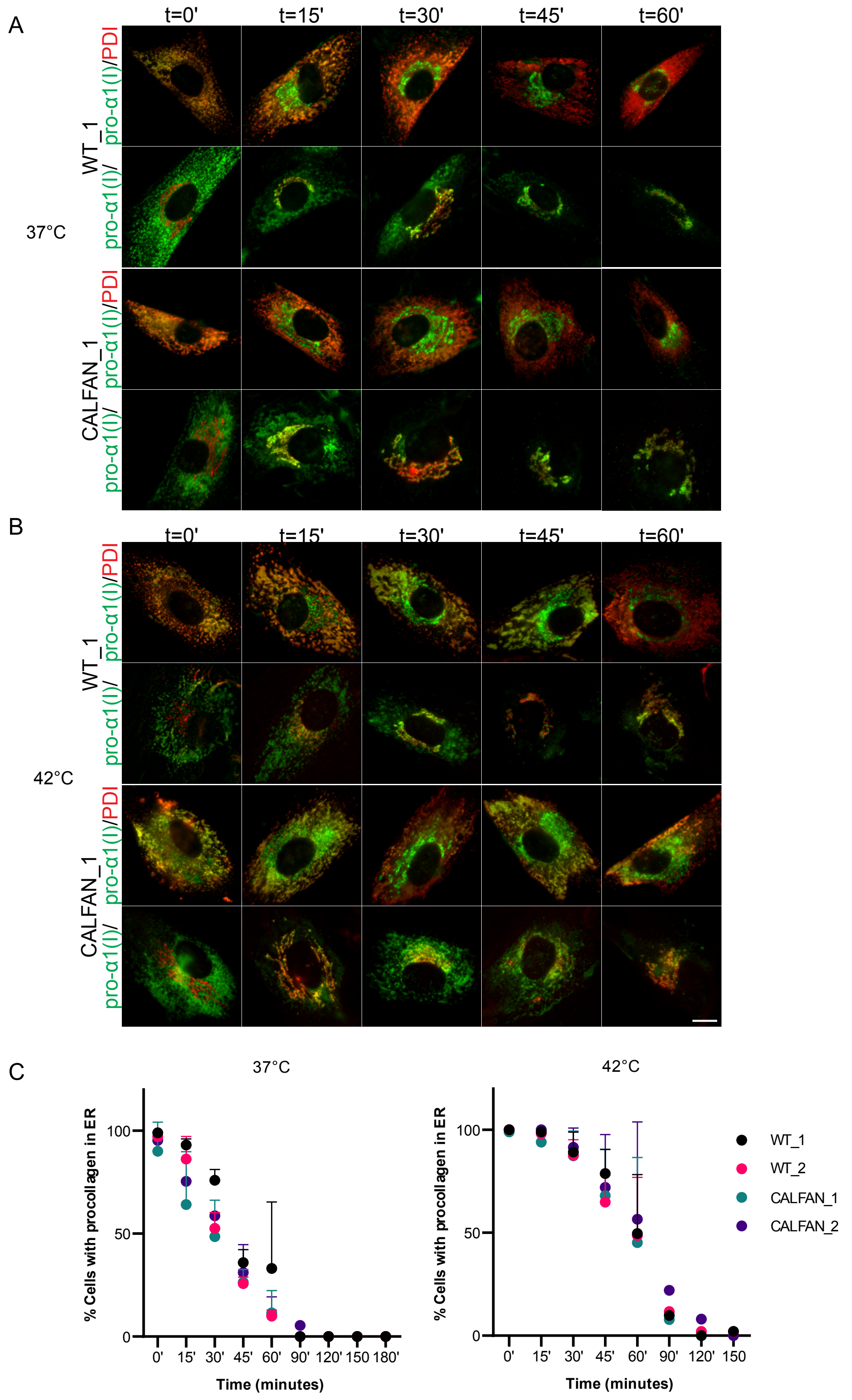

Fig. S4. Immunofluorescence analysis of procollagen trafficking in CALFAN fibroblasts. A,B). Wild-type (WT) and CALFAN fibroblasts were grown at 37°C or 42°C for 48 h prior to medium change and induction of traffic at either 37°C or 42°C in fresh medium containing ascorbic acid and cycloheximide. At the timepoints (t) indicted (t, in minutes) cells were fixed and labelled with antibodies against pro-α1(I) (green) and either PDI or GM130 (red). Scale bar, 10 µm. **C)** The percentage of wild-type and CALFAN cells with procollagen in the ER at each timepoint was calculated. Error bars represent mean with SD, n=2 independent experiments up to and including 60 minutes after which n=1. Multiple Mann-Whitney tests. Differences were found to be not significant except for 37°C, 45,' WT_1 vs CALFAN_1 where p=0.004 and adjusted p=0.02.

Disease Models & Mechanisms • Supplementary information

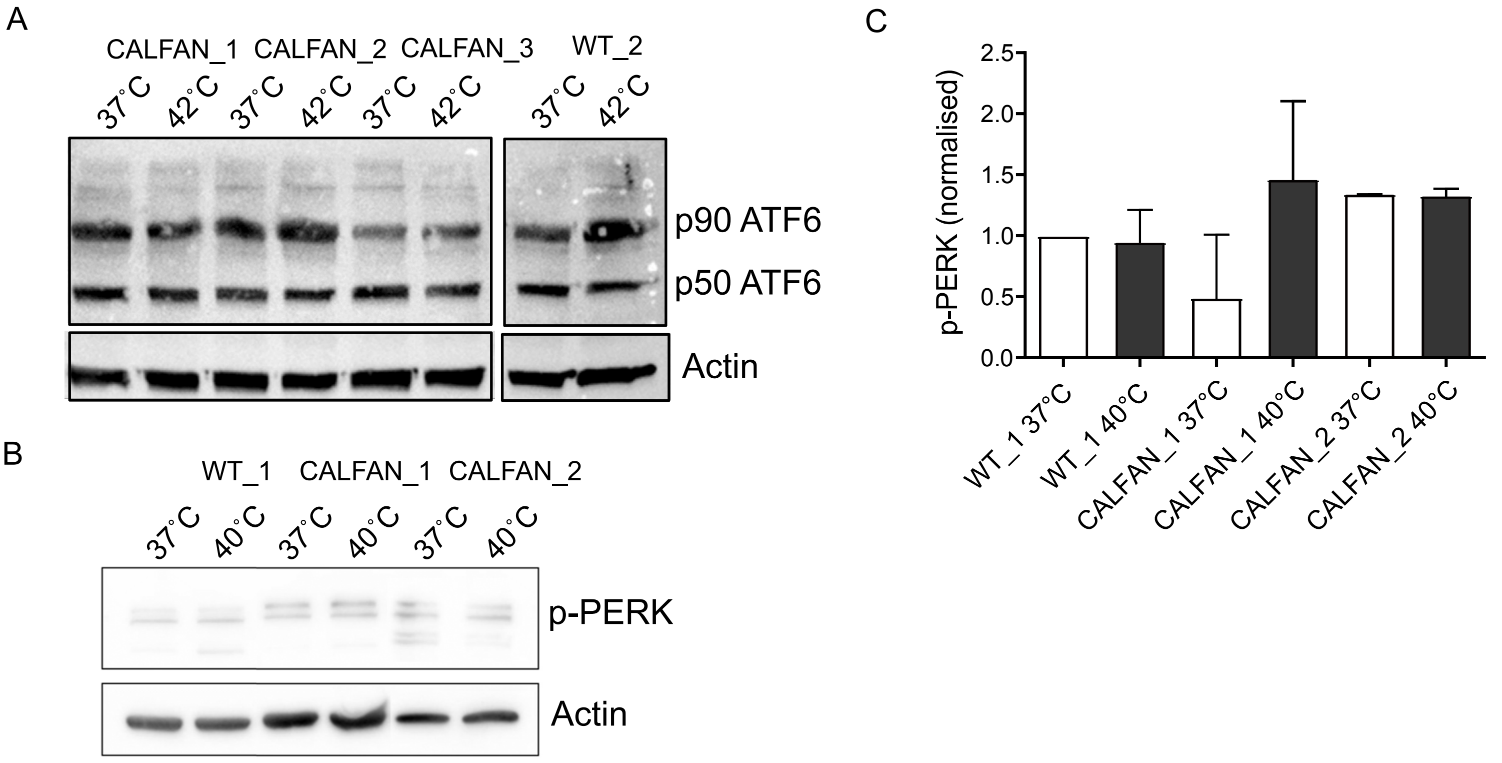

Fig. S5. Analysis of ATF6 and PERK in CALFAN fibroblasts. Wild-type and CALFAN fibroblasts were grown separately for two days 37°C in culture medium. For the final 24 hours cells were either kept at 37°C or moved to 40°C, cell lysates were generated and blotting was carried out using antibodies against ATF6 (full-length p90 ATF6, cleaved form p50 ATF6) **(A)** and phospho-PERK **(B)**, quantified as shown **(C)**. Actin was used as the loading control in all blots. Error bars represent mean with SD, n=3 independent experiments. Multiple unpaired t tests with Welch's correction. Differences were found to be not significant.

Table S1. Genotype and clinical phenotype of studied CALFAN patients. Genotype and individual phenotypic data regarding affected organ systems in CALFAN syndrome of the patients whose fibroblasts have been used within this study. CALFAN: low γ-glutamyl-transferase cholestasis, acute liver failure, and neurodegeneration; HP: human phenotype; N: no; Y: yes.

Patient identifier	Variant Allel 1 NM_020680.3	Variant allel 2 NM_020680.3	Publication	Patient number in publication	Affected organ systems	Abnormality of the liver (HP:0001392)	Abnormality of the nervous system (HP:0000707)	Abnormality of the skeletal system (HP:0000924)	Abnormality of the musculature (HP:0003011)	Growth abnormality (HP:0001507)	Abnormality of the endocrine system (HP:0000818)
CALFAN_1	c.1882C>T (Gln628*)	c.1882C>T p.(Gln628*)	Lenz et al., 2018	F1:II.2		Y	Y	Y	Y	N	N
CALFAN_2	c.1433A> G p.(Asp478Gly)	c.1433A> G p.(Asp478Gly)	Lenz et al., 2018	F2:II.5		Y	Y	N	N	N	N
CALFAN_4	c.169C>T p.(Gln57*)	c.169C>T p.(Gln57*)	Lenz et al., 2018	F4:II.1		Y	Y	Y	Y	N	N
CALFAN_5	c.314C>T p.(Ala105Val)	c.314C>T p.(Ala105Val)	Lenz et al., 2018	F5:II.3		Y	Y	N	N	N	N

Disease Models & Mechanisms • Supplementary information
